# On both magnetized and non-magnetized dual stratified medium via stream lines topologies: A generalized formulation

**DOI:** 10.1038/s41598-019-42726-5

**Published:** 2019-04-19

**Authors:** Khalil Ur Rehman, M. Y. Malik, Qasem M. Al-Mdallal, Mostafa Zahri

**Affiliations:** 1 Department of Mathematics, Air University, PAF Complex E-9, Islamabad, 44000 Pakistan; 2 Department of Mathematics, College of Sciences, King Khalid University, Abha, 61413 Saudi Arabia; 3 Department of Mathematical Sciences, United Arab Emirates University, P.O. Box 15551, Al Ain, Abu Dhabi United Arab Emirates; 4 College of Sciences, Department of Mathematics, Research Group MASEP, University of Sharjah, P.O Box 27272 Sharjah, United Arab Emirates

**Keywords:** Computational science, Nanoscale materials

## Abstract

The major concern of current pagination is to report the doubly stratified medium subject to both magnetized and non-magnetized flow fields. For this purpose both the Newtonian and non-Newtonian liquids are considered in a double stratified medium having magnetic field interaction. To be more specific, a generally accepted rheological liquid around a cylindrical surface having constant radius embedded in magnetized doubly stratified media is taken into account. Additionally, flow field is manifested with various pertinent physical effects. The flow problem statement is defended through generalized formulation via fundamental laws. A computational scheme is executed and stream lines topologies are constructed for the both magnetized and non-magnetized stratified medium to explore the interesting features. It is observed that the Casson fluid velocity towards cylindrical surface is higher in magnitude as compared to flat surface. Such observation is same for the both the magnetized and non-magnetized flow fields. Our general formulation yields some existing attempts in the literature. The variations in local skin friction coefficient (LSFC), local Nusselt number (LNN) and local Sherwood number (LSN) are provided with the aid of tabular forms. It is trusted that the obtain observations via stream lines topologies will serve a clear insight to the said flow problem.

## Introduction

The fluid flow having interaction with applied magnetic field is termed as magneto-hydrodynamic flow. The engaged fluid can be from Newtonian and non-Newtonian models. The Newtonian fluids possesses linear relation between shear stress and shear rate. This relation is well-known by Newton’s law of viscosity. It is named after Sir Issac Newton. The common examples subject to Newtonian are alcohol, mineral, gasoline and water to mention just a few. The non-Newtonian do not possesses linear relation between shear stress and shear rate. The non-Newtonian class can be explore by dividing into subclasses, namely *Dilatant* (when viscosity is increasing function of shear like in silly putty, quicksand, water and cornflour), *Pseudoplastic* (when viscosity is decreasing function of shear for instance in ketchup), Thixotropic (when viscosity is time dependent decreasing function of shear like in glue, paint, asphalt and cosmetics) and *Rheopectic* (when viscosity is time dependent increasing function of applied shear like cream and gypsum paste). The movement of electrical conductor (EC) in existing magnetic field results EMF (electromotive force). Such force namely EMF is direct relation with both strength of magnetic field and speed of moving EC. The generated EMF along with applied magnetic field yields a resistive force that opposes the motion of EC. As far as the study of the magnetohydrodynamics (MHD) is concern, the assumed fluid model will play the role of EC. To explore the importance of MHD flows scientists proposed the coupling of applied magnetic field by means of Naviers stokes equations and ultimate results predicts acceptable remarks regarding practical involvements in both industrial and engineering areas like plasma welding, liquid metals subject to casting processes, (EHCs) electrolytic Hall cells, shielding individualities and significant heat transfer characteristics are few examples. Owing the importance of magnetic field involvement various scientist and researchers shared their views like Hua and Walker^1^ discussed magnetic field interaction via rectangular ducts. In this problem an inclined applied magnetic field is taken into account. The recorded observations were presented with both analytical and numerical approaches. The impact of magnetic field on fluid flow due to flat surface was identified by Chaturvedi^2^. In this problem viscous fluid is considered as EC along with porous flat surface assumption while the induced magnetic effect is neglected in this attempt. Aldoss^3^ studied the effects of magnetic field by way of vertical cylinder. The non-Darcian model is assumed in porous medium. He found that both natural and forced convection regime have opposite results against applied magnetic field. The impact of magnetic field on unsteady flow regime is reported by Nanousis^4^. The incompressibility condition is implemented on viscous fluid being acted as an EC in this study. Moreover, he assumed oscillatory flat surface. Chamkha^5^ explored the effects of magnetic field subject to vertical surface. In this analysis a surface is considered in thermally stratified medium. Both the Halls and Hartmann impacts are reported numerically. Bhattacharyya and Gupta^6^ identified magnetic field individualities for stagnation point flow in a three dimensional frame. In this study they also entertained heat transfer properties. A viscous fluid is taken as an EC with arbitrary velocity gradients. The influence of magnetic field and the corresponding involvement namely Joule heating towards microplar fluid was taken by Hakiem *et al*.^7^. The micropolar fluid is taken as EC in this study. They found that both the SFC (skin friction coefficient) and HRT (heat transfer rate) reflects decline values for the positive values of magnetic field parameter. Mansour *et al*.^8^ addressed the effects of magnetic field in the presence of both heat and mass transfer characteristics. In this study they consider micropolar as an EC by way of circular cylinder. The impact of magnetic field along with variable viscosity was examined by Seddeek^9^. In this study heat transfer properties are considered in the presence of radiations. They found that the fluid velocity is decreasing function of magnetic field parameter but temperature profile is found to be increasing one. Amin^10^ considered micropolar fluid as an EC with constant suction assumption. The transverse magnetic field in assumed in this analysis. The impact of magnetic field was provided with the aid of both tables and graphs. The impact of magnetic field on an Oldroyd-B fluid was examined by Hayat *et al*.^11^. In this attempt Oldroy-B was taken as an EC and exact solution in this regard was presented. The magnetic interaction towards shrinking surface was discussed by Nadeem *et al*.^12^. In this work they considered Casson fluid as an EC and shrinking was exponentially proportional. Adomian decomposition method was utilized to report analytical solution and the outcomes in this regard were offered in terms of both tables and graphs. The Eying-Powell fluid was taken as an EC and magnetic characteristics were reported by Hayat *et al*.^13^. They presented series solution and graphical trends are provided to elaborate impact of magnetic field parameter. Since than many attempts were with respect MHD flows. The past and recent developments in this direction can be assessed in refs ^14–31^.

The strength of current pagination is generalized mathematical formulation and interpretation in both magnetized and non-magnetized frames. In this article Casson fluid is taken as an EC with doubly stratified medium. The Casson fluid flow regime is further manifested with various physical effects namely stagnation point, applied magnetic field, mixed convetion, thermal radiation, heat generation/absorption, Joule heating, chemical reaction and suspended nanoparticles. It is noticed that the mutual mathematical modelling subject to flow narrating differential equations that is Navier-Stokes equations and Maxwell equations generates complex structures and it seems impossible to obtain exact solutions. Therefore a computational algorithm is developed to report numerical results in terms of both tables and graphs. To be more specific, *Section-1* is devoted to skimmed the limited literature survey regarding magneto-hydrodynamic boundary layer flows which includes both Newtonian and non-Newtonians models. The generalized mathematical treatment for Casson fluid model is structured in *Section-2*. The detail analysis after implementation of computational algorithm is discussed in *Section-3*. In addition, *Section-3* is further divided into sub sections (*key to the graphs, key to the tables* etc.) to facilitate readers. The stream lines patterns for both magnetized and non-magnetized cases are provided as a graphical outcomes in *Section-4*. The sub cases subject to said problem are retraced and discussed in *Section-5*. The summary of analysis is offered as an itemized points in *Section-6*. An adopted procedure is trustful and it can be extended to some useful fluid models like Tangent hyperbolic fluid model, Williamson fluid model, Powell-Eyring fluid model, Jeffrey fluid model etc.

## Generalized Formulation

The flow model for the Casson fluid is mathematically modelled when various effects are taken into account namely, mixed convection, stagnation point, magnetic field, Joule heating, nonlinear radiations, heat generation/absorption, nanoparticles, temperature stratification, concentration stratification and chemical reaction. The fluid flow is achieved due to an inclined stretching cylindrical surface. For clarity the axial cylinder line is supposed to be parallel to *x*-axis and the perpendicular axis to the *x*-axis is taken as r-axis (radial direction). Through generally accepted mathematical laws, one can obtained the following flow narrating differential equations:

1 $$\frac{\partial (r\bar{u})}{\partial x}+\frac{\partial (r\bar{v})}{\partial r}=0,$$

2 $$\begin{array}{rcl}\bar{u}\frac{\partial \bar{u}}{\partial x}+\bar{v}\frac{\partial \bar{u}}{\partial r} & = & \nu (1+\frac{1}{\beta })(\frac{{\partial }^{2}\bar{u}}{\partial {r}^{2}}+\frac{1}{r}\frac{\partial \bar{u}}{\partial r})+{\bar{u}}_{e}\frac{\partial {\bar{u}}_{e}}{\partial x}\\  &  & -\,\frac{\sigma {{B}_{0}}^{2}}{\rho }(\bar{u}-{\bar{u}}_{e})+g{\beta }_{T}(\bar{T}-{\bar{T}}_{\infty })\,\cos \,\alpha \\  &  & +\,g{\beta }_{c}(\bar{C}-{\bar{C}}_{\infty })\,\cos \,\alpha ,\end{array}$$

3 $$\begin{array}{rcl}\bar{u}\frac{\partial \bar{T}}{\partial x}+\bar{v}\frac{\partial \bar{T}}{\partial r} & = & \frac{{\alpha }^{\cdot }}{r}\frac{\partial }{\partial r}(r\frac{\partial \bar{T}}{\partial r})+\tau (\frac{{D}_{T}}{{\bar{T}}_{\infty }}{(\frac{\partial \bar{T}}{\partial r})}^{2}+{D}_{B}\frac{\partial \bar{T}}{\partial r}\frac{\partial \bar{C}}{\partial r})\\  &  & -\,\frac{1}{\rho {c}_{p}r}\frac{\partial }{\partial r}(r{q}_{r})+\frac{{Q}_{0}}{{c}_{p}\rho }(\bar{T}-{\bar{T}}_{\infty })+\frac{\sigma {{B}_{o}}^{2}{\bar{u}}^{2}}{\rho {c}_{p}},\end{array}$$

4 $$\begin{array}{rcl}\bar{u}\frac{\partial \bar{C}}{\partial x}+\bar{v}\frac{\partial \bar{C}}{\partial r} & = & {D}_{B}(\frac{1}{r}\frac{\partial \bar{C}}{\partial r}+\frac{{\partial }^{2}\bar{C}}{\partial {r}^{2}})+\frac{{D}_{T}}{{\bar{T}}_{\infty }}(\frac{1}{r}\frac{\partial \bar{T}}{\partial r}+\frac{{\partial }^{2}\bar{T}}{\partial {r}^{2}})\\  &  & -\,{R}_{0}(\bar{C}-{\bar{C}}_{\infty }),\end{array}$$

after using the Roseland radiative heat flux that is ${q}_{r}=-\,(\frac{4}{3})\frac{{\sigma }^{\cdot }}{{k}^{\cdot }}\frac{\partial {\bar{T}}^{4}}{\partial r}$, one can conclude the modification:

5 $$\begin{array}{rcl}\bar{u}\frac{\partial \bar{T}}{\partial x}+\bar{v}\frac{\partial \bar{T}}{\partial r} & = & \frac{{\alpha }^{\cdot }}{r}\frac{\partial }{\partial r}(r\frac{\partial \bar{T}}{\partial r})+\tau (\frac{{D}_{T}}{{\bar{T}}_{\infty }}{(\frac{\partial \bar{T}}{\partial r})}^{2}+{D}_{B}\frac{\partial \bar{T}}{\partial r}\frac{\partial \bar{C}}{\partial r})\\  &  & +\,\frac{1}{\rho {c}_{p}r}(\frac{4}{3})\frac{{\sigma }^{\cdot }}{{k}^{\cdot }}\frac{\partial }{\partial r}(r\frac{\partial {\bar{T}}^{4}}{\partial r})\\  &  & +\,\frac{{Q}_{0}}{{c}_{p}\rho }(\bar{T}-{\bar{T}}_{\infty })+\frac{\sigma {{B}_{o}}^{2}{\bar{u}}^{2}}{\rho {c}_{p}},\end{array}$$

with end point conditions:

6 $$\begin{array}{c}\bar{u}=U(x)=ax,\,\bar{v}=0,\,{\rm{at}}\,r=R,\,\bar{u}\to {\bar{u}}_{e}={a}^{\ast }x,\,{\rm{when}}\,r\to \infty ,\\ \bar{T}(x,r)={\bar{T}}_{w}(x)={\bar{T}}_{0}+\frac{bx}{L},\bar{C}(x,r)={\bar{C}}_{w}(x)={\bar{C}}_{0}+\frac{dx}{L}\,{\rm{at}}\,r=R,\\ \bar{T}(x,r)\to {\bar{T}}_{\infty }(x)={\bar{T}}_{0}+\frac{cx}{L},\bar{C}(x,r)\to {\bar{C}}_{\infty }(x)={\bar{C}}_{0}+\frac{ex}{L}\,{\rm{as}}\,r\to \infty ,\end{array}$$

since the mathematical problem given in Eqs (1–5) with an endpoint conditions by Eq. (6) is highly non-linear therefore to report an exact solution seems impossible. Therefore, to seek the numerical solution we need an equivalent system of ordinary differential equations. It can be attained by using set of similarity transformation. This approach is scientifically and mathematically proven and considered by various prolific investigators like Sakiadis, Blasius, Prandtl and up-to today, see refs^32–34^. One of application of Lie symmetry analysis is order reduction towards differential equations. Ferdows *et al*.^35^ carried Lie symmetry approach to find set of similarity transformation for conversion of partial differential equations into ordinary differential system. Some recent trustful attempts subject to similarity transformation can assessed in refs^36–42^. Our attention is to solve the system of ordinary differential equations, therefore the widely used set of transformation^37–42^ for the cylindrical surface is given as

7 $$\begin{array}{c}\bar{u}=\frac{{U}_{0}x}{L}F^{\prime} (\eta ),\,\bar{v}=-\,\frac{R}{r}\sqrt{\frac{{U}_{0}v}{L}}F(\eta ),\,\eta =\frac{{r}^{2}-{R}^{2}}{2R}{(\frac{{U}_{0}}{vL})}^{\frac{1}{2}},\,\psi ={(\frac{{U}_{0}v{x}^{2}}{L})}^{\frac{1}{2}}RF(\eta ),\\ C(\eta )=\frac{\bar{C}-{\bar{C}}_{\infty }}{{\bar{C}}_{w}-{\bar{C}}_{0}},\,T(\eta )=\frac{\bar{T}-{\bar{T}}_{\infty }}{{\bar{T}}_{w}-{\bar{T}}_{0}},\end{array}$$

the velocity relations towards stream function are written by form:

8 $$\bar{u}=\frac{1}{r}(\frac{\partial \psi }{\partial r}),\,\bar{v}=-\,\frac{1}{r}(\frac{\partial \psi }{\partial x}),$$

use of Eq. (8) gives:

9 $$\begin{array}{l}(1+2K\eta )\frac{{d}^{3}F(\eta )}{d{\eta }^{3}}+2K\frac{{d}^{2}F(\eta )}{d{\eta }^{2}}+\frac{1}{\beta }((1+2K\eta )\frac{{d}^{3}F(\eta )}{d{\eta }^{3}}+2K\frac{{d}^{2}F(\eta )}{d{\eta }^{2}})\\ \,\,\,+\,F(\eta )\frac{{d}^{2}F(\eta )}{d{\eta }^{2}}-{(\frac{dF(\eta )}{d\eta })}^{2}-{\gamma }^{2}(\frac{dF(\eta )}{d\eta }-A)+{A}^{2}+{\lambda }_{m}(T(\eta )+NC(\eta ))\,\cos \,\alpha =0,\end{array}$$

10 $$\begin{array}{l}(1+2K\eta )(1+\frac{4}{3}{R}_{T})\frac{{d}^{2}T(\eta )}{d{\eta }^{2}}+2K(1+\frac{4}{3}{R}_{T})\frac{dT(\eta )}{d\eta }+{\rm{\Pr }}\,Nb(1\\ \,\,\,+\,2K\eta )(\frac{dT(\eta )}{d\eta }\frac{dC(\eta )}{d\eta }){\rm{\Pr }}(F(\eta )\frac{dT(\eta )}{d\eta }-\frac{dF(\eta )}{d\eta }{\delta }_{1}\\ \,\,\,+\,QT(\eta )-\frac{dF(\eta )}{d\eta }T(\eta )+Ec{\gamma }^{2}F{(\eta )}^{2})\\ \,\,\,+\,{\rm{\Pr }}\,Nb(1+2K\eta )(\frac{Nt}{Nb}{(\frac{dT(\eta )}{d\eta })}^{2})+=0,\end{array}$$

11 $$\begin{array}{c}(1+2K\eta )(\frac{{d}^{2}C(\eta )}{d{\eta }^{2}}+\frac{Nt}{Nb}\frac{{d}^{2}T(\eta )}{d{\eta }^{2}})+{\rm{\Pr }}\,Le(F(\eta )\frac{dC(\eta )}{d\eta }-\frac{dF(\eta )}{d\eta }C(\eta )-\frac{dF(\eta )}{d\eta }{\delta }_{2})\\ \,\,\,+\,2K(\frac{dC(\eta )}{d\eta }+\frac{Nt}{Nb}\frac{dT(\eta )}{d\eta })-{R}_{c}C(\eta )=0,\end{array}$$

with transformed conditions:

12 $$\begin{array}{c}\frac{dF(\eta )}{d\eta }=1,\,\,F(\eta )=0,\,\,T(\eta )=1-{\delta }_{1},\,\,\,C(\eta )=1-{\delta }_{2},\,{\rm{at}}\,\eta =0,\\ \frac{dF(\eta )}{d\eta }\to A,\,\,T(\eta )\to 0,\,\,C(\eta )\to 0,\,\,{\rm{when}}\,\,\eta \to \infty .\end{array}$$

The involved parameters are defined as below:

13 $$\begin{array}{c}K=\tfrac{1}{R}\sqrt{\frac{v}{a}},\,a=\tfrac{{U}_{0}}{L},\,\gamma =\sqrt{\tfrac{\sigma {{B}_{0}}^{2}}{\rho a},}\,A=\tfrac{{a}^{\ast }}{a},\,{\lambda }_{m}=\tfrac{Gr}{{{\mathrm{Re}}_{x}}^{2}},\,N=\tfrac{G{r}^{\cdot }}{Gr},\,{R}_{T}=\tfrac{4{\sigma }^{\cdot }{{T}^{3}}_{\infty }}{{k}^{\cdot }k},\\ {\rm{\Pr }}=\tfrac{v}{{\alpha }^{\cdot }},\,Nb=\tfrac{\tau {D}_{B}({\bar{C}}_{w}-{\bar{C}}_{\infty })}{v},\,Nt=\tfrac{\tau {D}_{T}({\bar{T}}_{w}-{\bar{T}}_{\infty })}{v{\bar{T}}_{\infty }},\,{\delta }_{1}=\tfrac{c}{b},\,Q=\tfrac{L{Q}_{0}}{{U}_{0}\rho {c}_{p}},\,Le=\tfrac{{\alpha }^{\cdot }}{{D}_{B}},\\ {\delta }_{2}=\tfrac{e}{d},\,{R}_{c}=\tfrac{{R}_{0}L}{{U}_{0}},\,Gr=\tfrac{g{\beta }_{T}({\bar{T}}_{w}-{\bar{T}}_{0}){x}^{3}}{{v}^{2}},\,G{r}^{\cdot }=\tfrac{g{\beta }_{C}({\bar{C}}_{w}-{\bar{C}}_{0}){x}^{3}}{{v}^{2}},\,Ec=\tfrac{{U}_{0}x}{{c}_{p}({\bar{T}}_{w}-{\bar{T}}_{\infty })L},\end{array}$$

and the local skin friction coefficient at the surface of cylinder is given by:

14 $${C}_{f}=\frac{{\tau }_{w}}{\rho \frac{{U}^{2}}{2}},\,{\tau }_{w}=\mu (1+\frac{1}{\beta }){(\frac{\partial u}{\partial r})}_{r=R},$$

in dimensionless practice, it is written as:

15 $$0.5{C}_{f}\sqrt{{{\rm{Re}}}_{x}}=(1+\frac{1}{\beta })F^{\prime\prime} (0),$$

with ${{\rm{Re}}}_{x}=\frac{{U}_{0}{x}^{2}}{vL}$ be the local Reynolds number. The expression for the both local Nusselt and the local Sherwood number**s** are given as:

16 $$\begin{array}{c}N{u}_{x}=\frac{x{q}_{w}}{k({\bar{T}}_{w}-{\bar{T}}_{0})},\,{q}_{w}=-\,k{(\frac{\partial \bar{T}}{\partial r})}_{r=R}+{({q}_{r})}_{r=R},\\ Sh=\frac{x{j}_{w}}{D({\bar{C}}_{w}-{\bar{C}}_{0})},\,{j}_{w}=-\,D{(\frac{\partial \bar{C}}{\partial r})}_{r=R},\end{array}$$

the dimensionless form of these expression are pre-arranged as:

17 $$\begin{array}{rcl}\frac{N{u}_{x}}{\sqrt{{{\rm{Re}}}_{x}}} & = & -\,(1+\frac{4}{3}{R}_{d})T^{\prime} (\eta ),\,\,{\rm{as}}\,\,\eta \to 0,\\ \frac{S{h}_{x}}{\sqrt{{{\rm{Re}}}_{x}}} & = & -\,C^{\prime} (\eta ),\,\,{\rm{as}}\,\,\eta \to 0.\end{array}$$

For the numerical solution the shooting method with Runge-Kutta algorithm is utilized. The complete description in this regard is provided by way of Fig. 2.

## Analysis

### Key to the graphs

**Figure 1:**
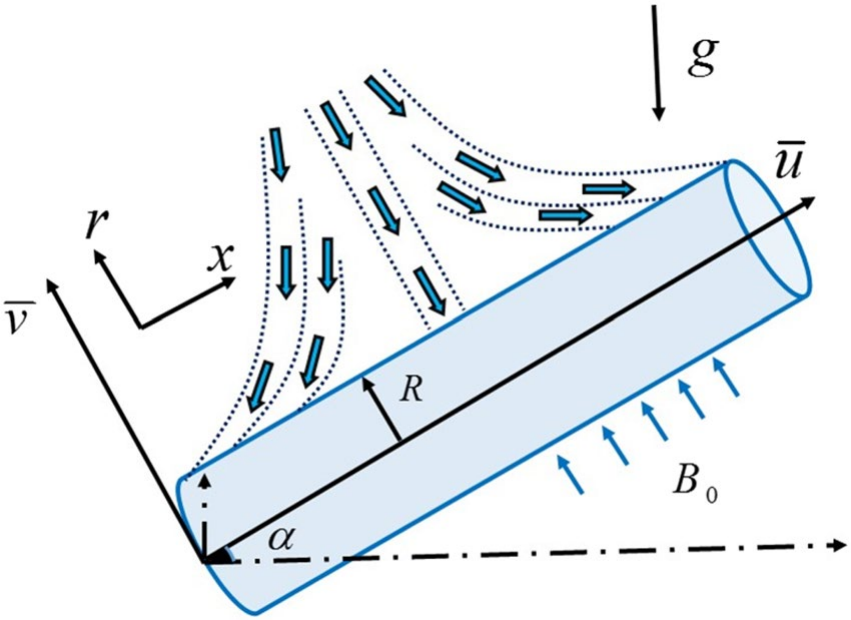
Problem schematic diagram.

**Figure 2:**
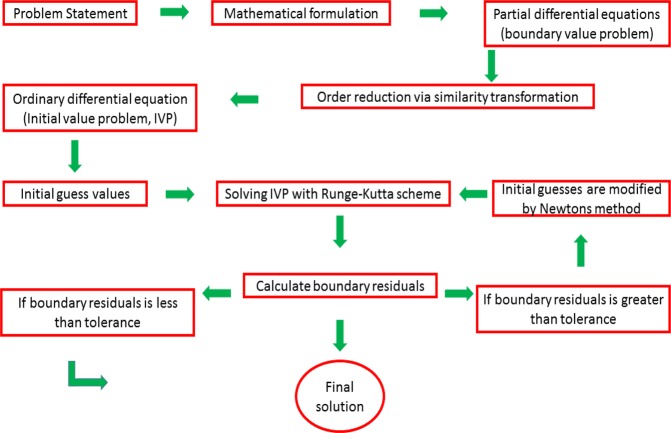
Schematic diagram of numerical scheme.

**Figure 3:**
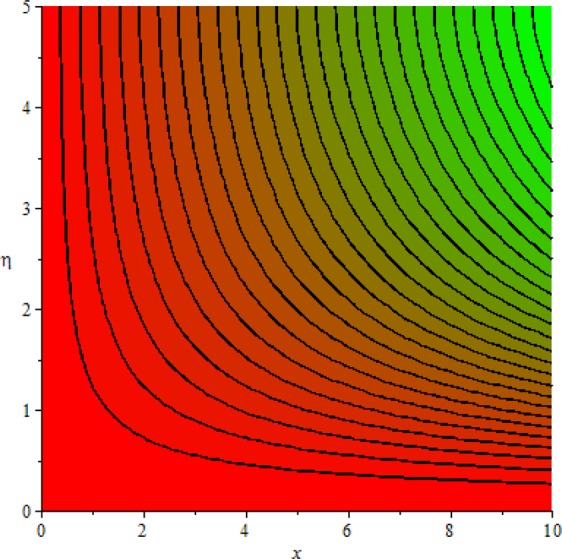
Stream lines when *γ* = 0.5 and *K* = 0.7.

**Figure 4:**
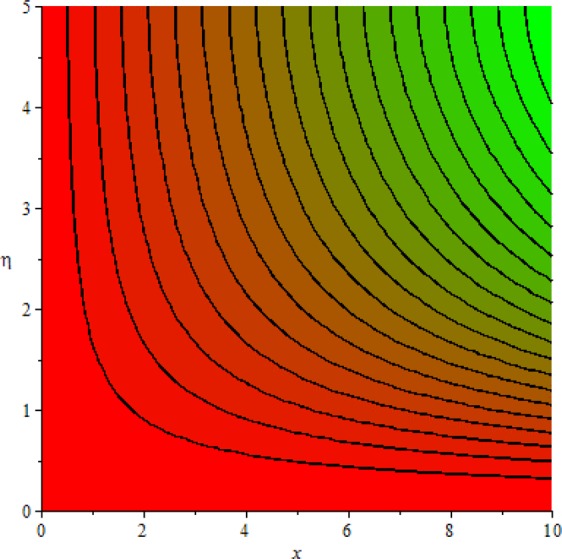
Stream lines when *γ* = 0.5 and *K* = 0.0.

**Figure 5:**
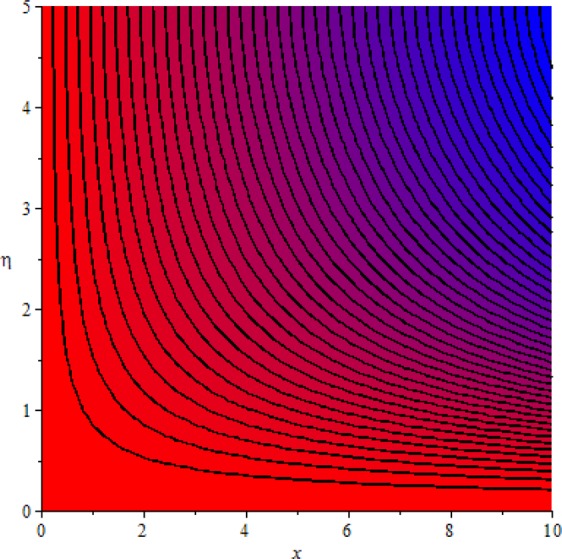
Stream lines when *γ* = 0.0 and *K* = 0.7.

**Figure 6:**
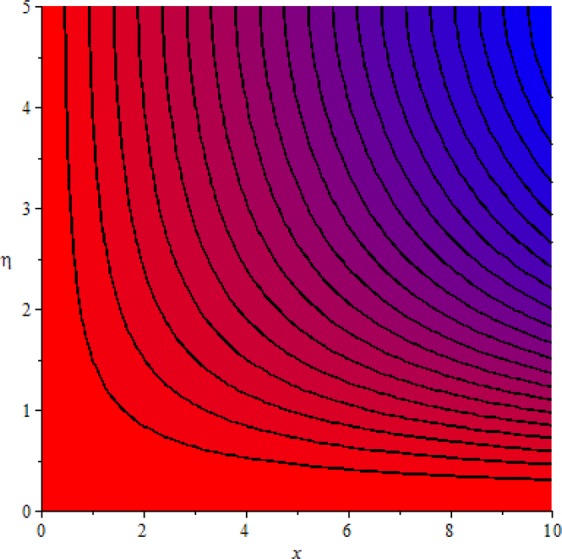
Stream lines when *γ* = 0.0 and *K* = 0.0.

**Figure 7:**
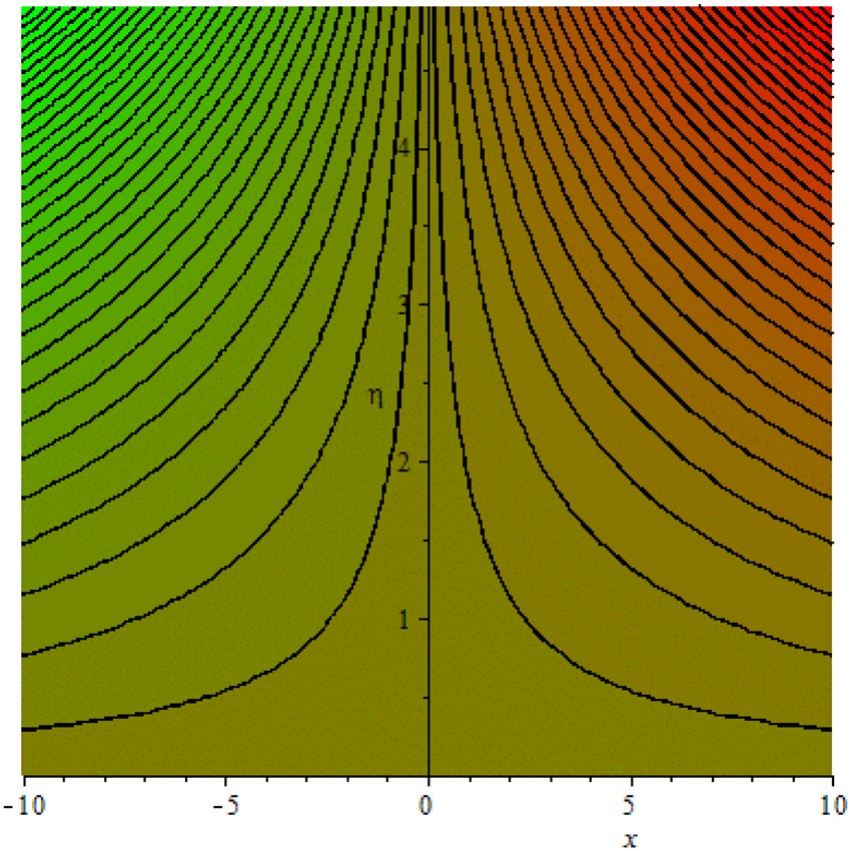
Stream lines when *γ* = 0.0 and *A* = 0.5.

**Figure 8:**
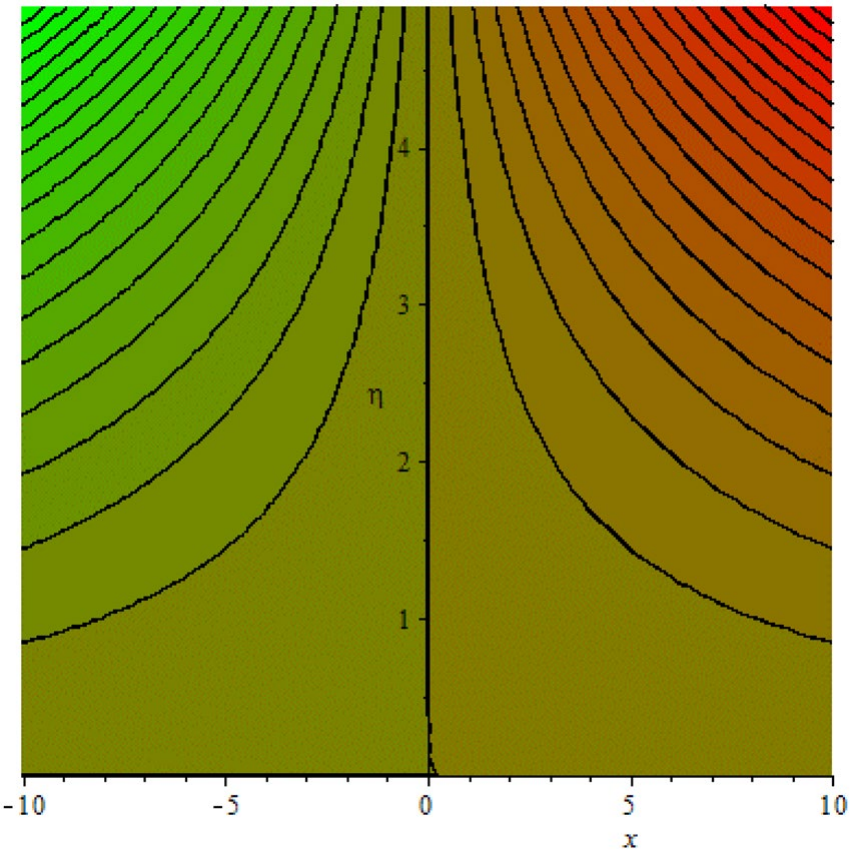
Stream lines when *γ* = 0.5 and *A* = 0.5.

**Figure 9:**
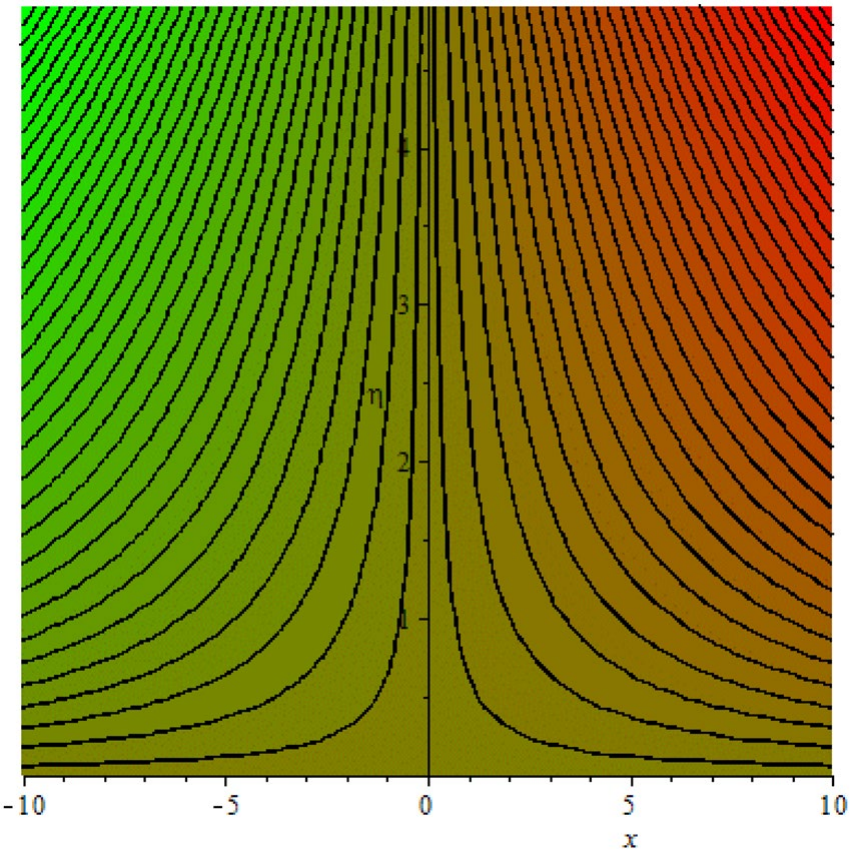
Stream lines when *γ* = 0.0 and *A* = 1.0.

**Figure 10:**
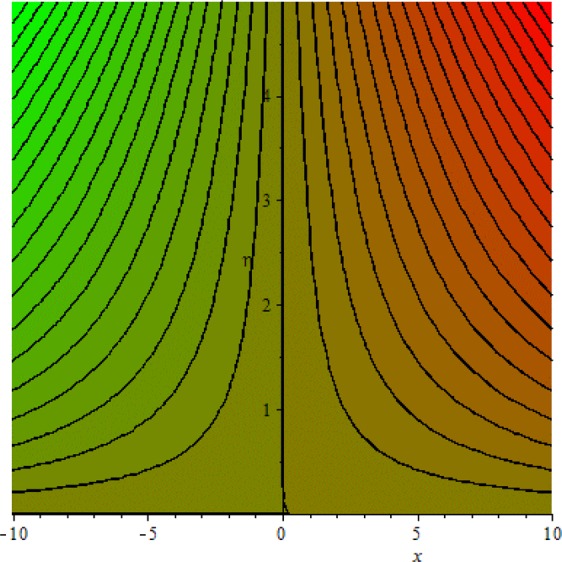
Stream lines when *γ* = 0.5 and *A* = 1.0.

**Figure 11:**
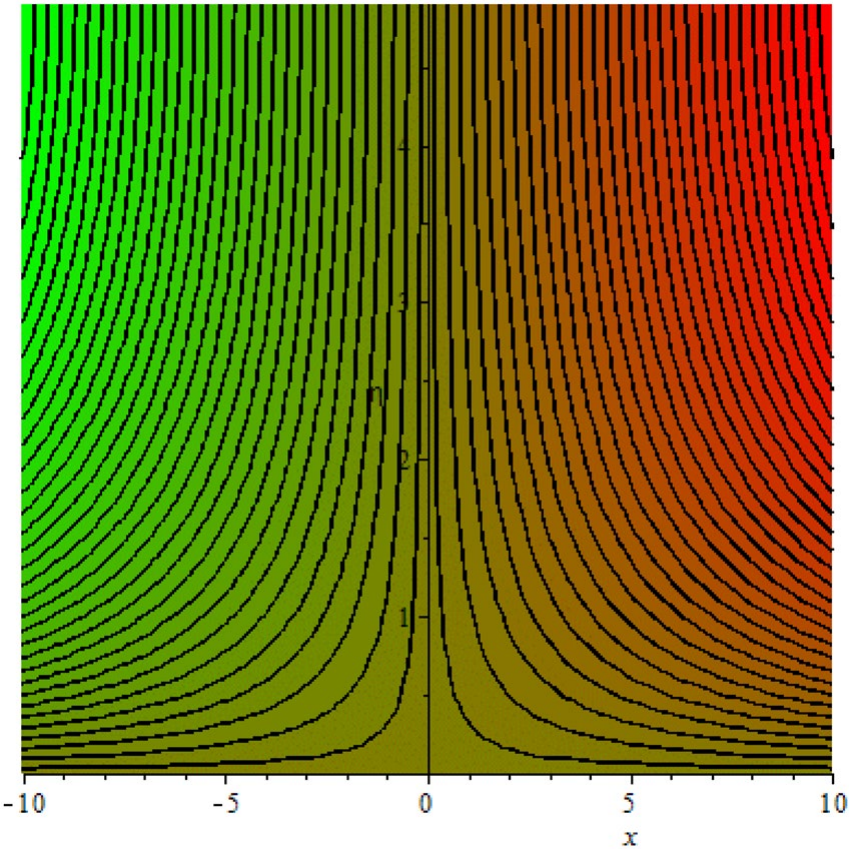
Stream lines when *γ* = 0.0 and *A* = 1.5.

**Figure 12:**
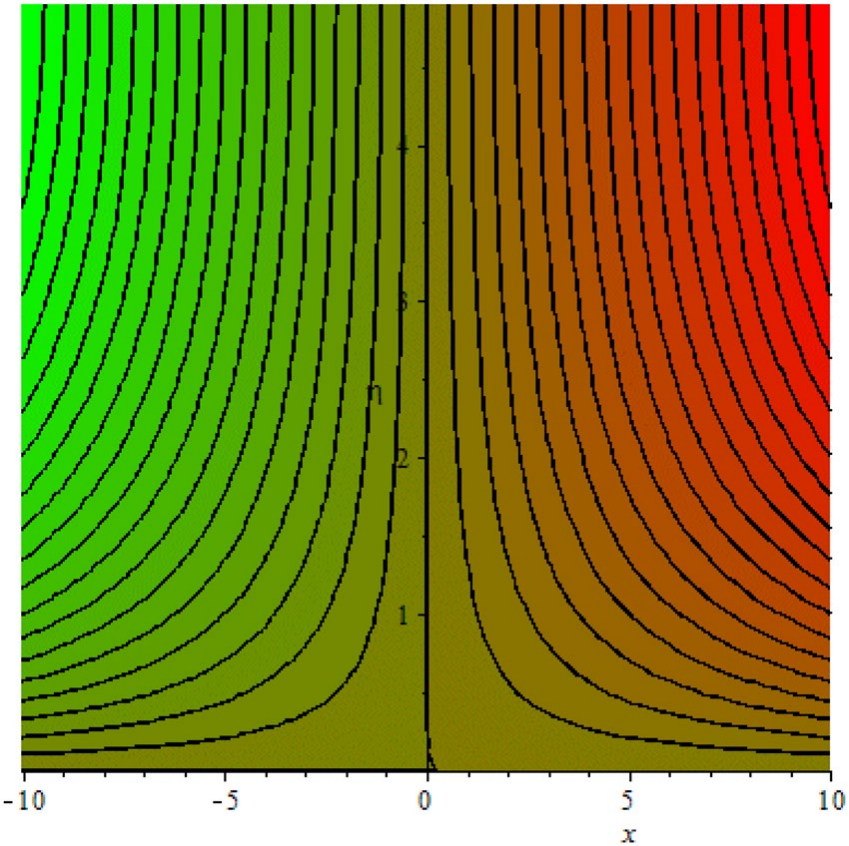
Stream lines when *γ* = 0.5 and *A* = 1.5.

**Figure 13:**
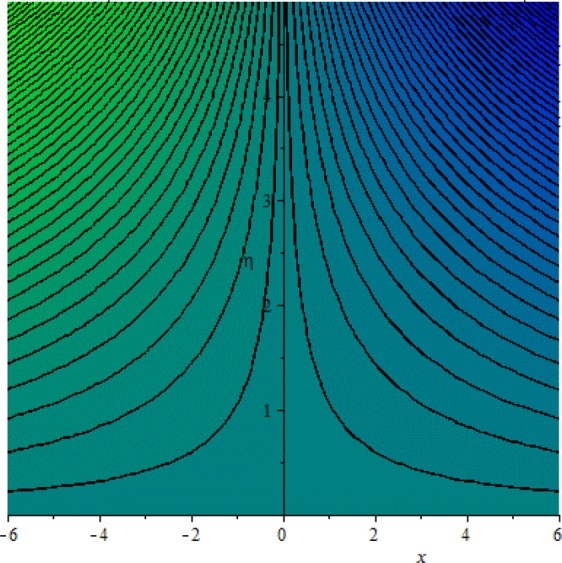
Stream lines when *γ* = 0.0 and *β* = 1.3.

**Figure 14:**
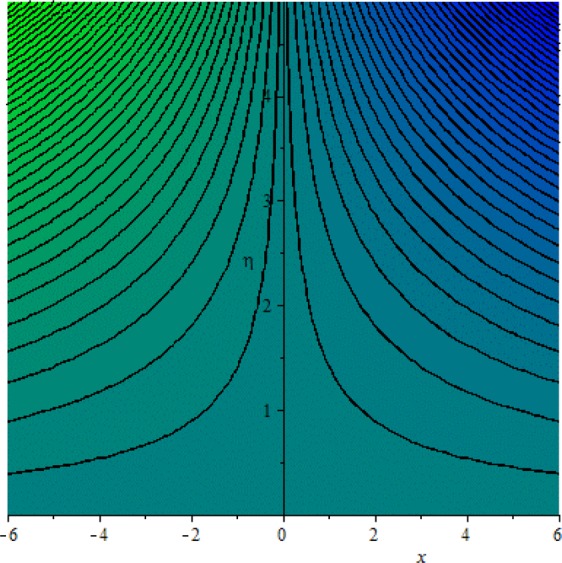
Stream lines when *γ* = 0.5 and *β* = 1.3.

**Figure 15:**
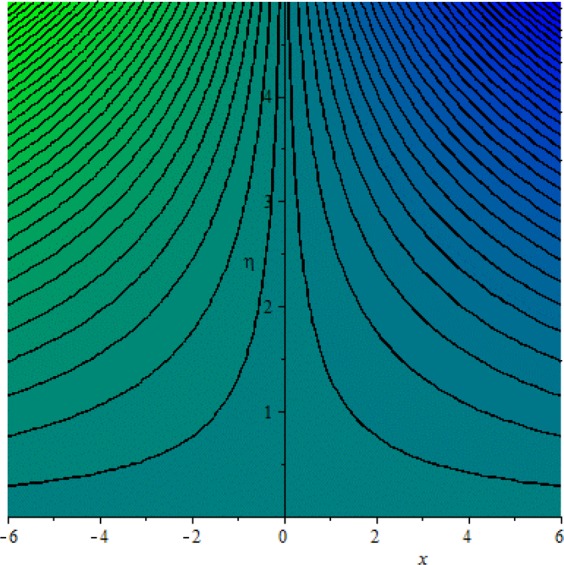
Stream lines when *γ* = 0.0 and *β* → ∞.

**Figure 16:**
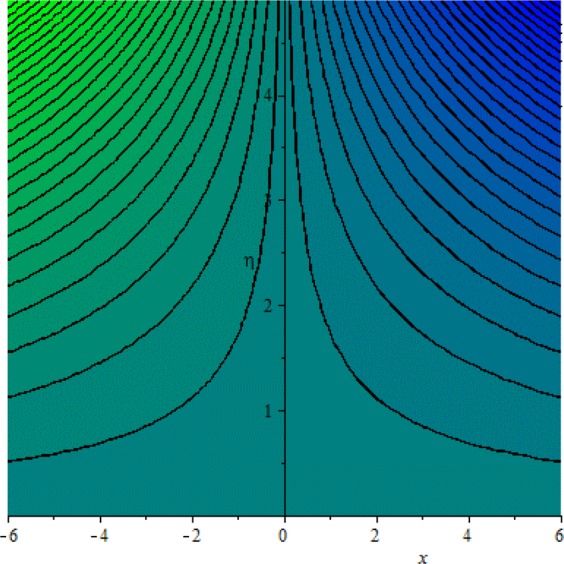
Stream lines when *γ* = 0.5 and *β* → ∞.

**Figure 17:**
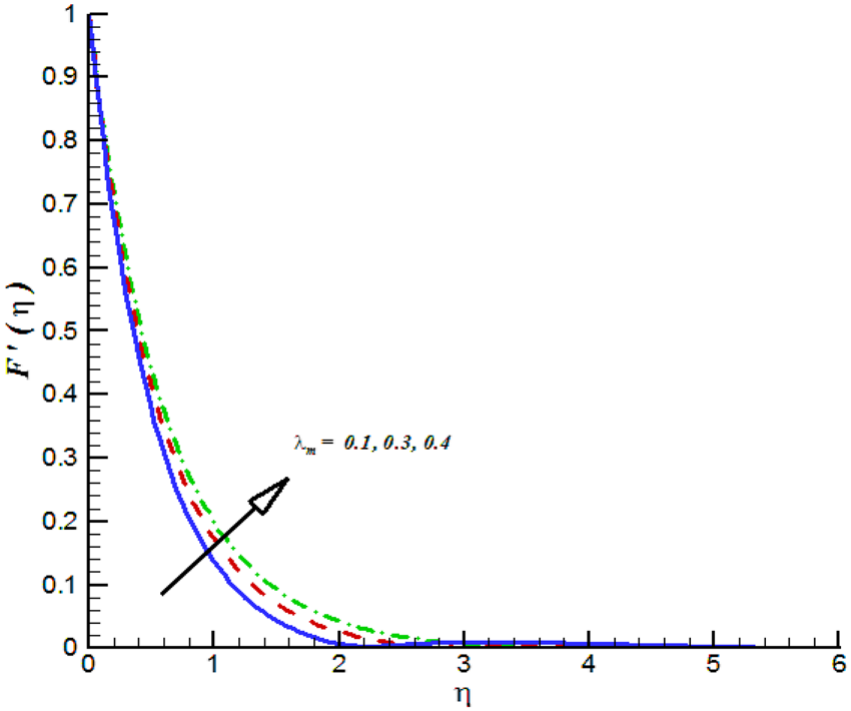
Impact of *λ*_*m*_ on *F*′(*η*).

**Figure 18:**
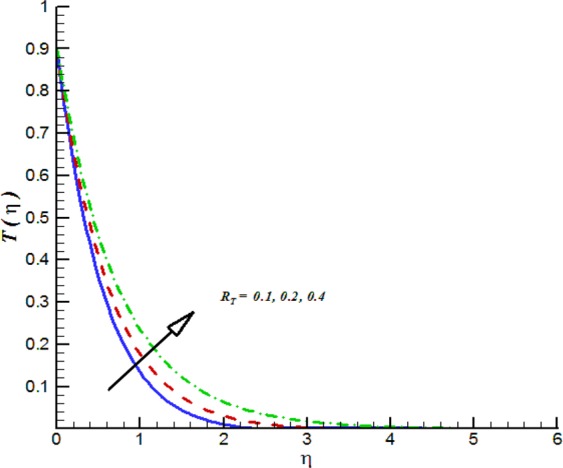
Impact of *R*_*T*_ on *T*(*η*).

**Figure 19:**
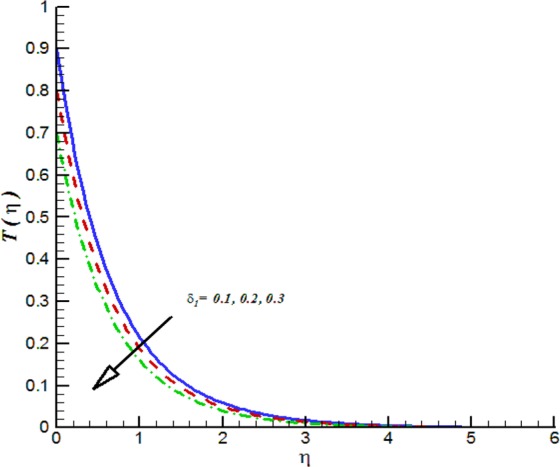
Impact of *δ*_1_ on *T*(*η*).

**Figure 20:**
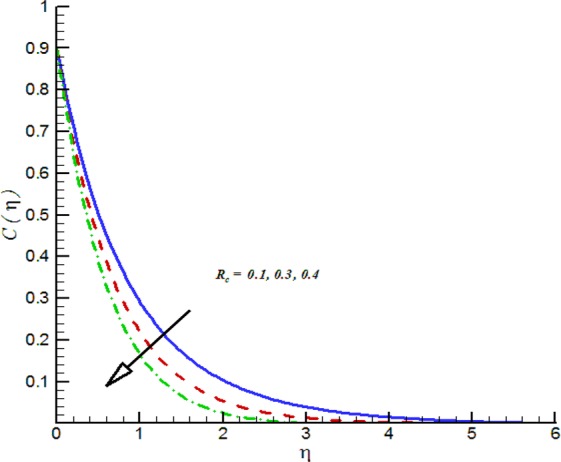
Impact of *R*_*c*_ on *C*(*η*).

Figures 3–20 are plotted to examine the flow field pattern of Casson liquid in both magnetized (*γ* ≠ 0) and non-magnetized (*γ* = 0) double stratified medium (*δ*_1_ ≠ 0, *δ*_2_ ≠ 0). Figure 1 provides the flow illustration of present problem. Figure 2 provides schematic diagram of numerical scheme. Figures 3 and 4 depicts stream lines pattern of Casson fluid in magnetized stratified medium for both flat (*K* = 0) and cylinder geometry (*K* ≠ 0) while Figs 5 and 6 are plotted against non-magnetized stratified medium subject to Casson liquid for both flat and cylinder geometry. It is important to note that Fig. 3–6 are examine without the assumption of stagnation point (*A* = 0) but Figs 7–16 are provided in the presence of stagnation point (*A* ≠ 0). In detail, Figs 7–12 are plotted for (*A* < 1), (*A* = 1) and (*A* > 1) respectively. Lastly, Figs 13 and 14 offered Casson fluid model stream lines topologies while Figs 15 and 16 depicts viscous fluid model (*β* → ∞). Figures 17–20 are provided to offer the variations in fluid velocity, temperature and nano-concentration profiles towards flow controlling parameters.

### Key to the tables

Tables 1–8 are constructed to offer the variations in local skin friction coefficient (LSFC), local Nusselt number (LNN) and local Sherwood number (LSN) via curvature parameter (*K*), Casson fluid parameter (*β*), mixed convection parameter (*λ*_*m*_), Prandtl number (Pr), heat generation parameter (*Q*^+^), temperature stratification parameter (*δ*_1_), Schmidt number (*Sc*), concentration stratification parameter (*δ*_2_), chemical reaction parameter (*R*_*c*_) and Lewis number (*Le*). To be more specific, Tables 1, 3 and 6 reports the variations in LSFC. Further, Tables 2, 4 and 7 offer variations in LNN while the numerical values towards various parameters for LSN is reported in Tables 5 and 7.

**Table 1:** LSFC comparison with ref.^31^.

*K*	*β*	*λ*_*m*_	Pr	*δ*_1_	*Q* ^+^	ref.^31^		Present Outcomes	
						*F″*(0)	0.5*C*_*F*_$\sqrt{{\bf{R}}{{\bf{e}}}_{{\boldsymbol{x}}}}$	*F″*(0)	0.5*C*_*F*_$\sqrt{{\bf{R}}{{\bf{e}}}_{{\boldsymbol{x}}}}$
0.1	0.1	0.1	0.1	0.1	0.1	−0.7652	−0.3826	−0.7652	−0.3826
0.2	0.1	0.1	0.1	0.1	0.1	−0.8073	−0.4036	−0.8073	−0.4036
0.3	0.1	0.1	0.1	0.1	0.1	−0.8497	−0.4248	−0.8497	−0.4248
0.1	1.3	0.1	0.1	0.1	0.1	−0.7652	−0.3826	−0.7652	−0.3826
0.1	1.4	0.1	0.1	0.1	0.1	−0.7760	−0.3884	−0.7760	−0.3884
0.1	1.5	0.1	0.1	0.1	0.1	−0.7857	−0.3928	−0.7857	−0.3928
0.1	0.1	0.1	0.1	0.1	0.1	−0.7652	−0.3826	−0.7652	−0.3826
0.1	0.1	0.2	0.1	0.1	0.1	−0.7358	−0.3679	−0.7358	−0.3679
0.1	0.1	0.3	0.1	0.1	0.1	−0.7071	−0.3535	−0.7071	−0.3535
0.1	0.1	0.1	0.6	0.1	0.1	−0.7633	−0.3816	−0.7633	−0.3816
0.1	0.1	0.1	0.7	0.1	0.1	−0.7652	−0.3826	−0.7652	−0.3826
0.1	0.1	0.1	0.8	0.1	0.1	−0.7670	−0.3835	−0.7670	−0.3835
0.1	0.1	0.1	0.1	0.1	0.1	−0.7652	−0.3826	−0.7652	−0.3826
0.1	0.1	0.1	0.1	0.2	0.1	−0.7703	−0.3851	−0.7703	−0.3851
0.1	0.1	0.1	0.1	0.3	0.1	−0.7754	−0.3877	−0.7754	−0.3877
0.1	0.1	0.1	0.1	0.1	0.1	−0.7652	−0.3826	−0.7652	−0.3826
0.1	0.1	0.1	0.1	0.1	0.2	−0.7640	−0.3821	−0.7640	−0.3821
0.1	0.1	0.1	0.1	0.1	0.3	−0.7626	−0.3813	−0.7626	−0.3813

**Table 2:** LNN comparison with ref.^31^.

*K*	*β*	*λ*_*m*_	Pr	*δ*_1_	*Q* ^+^	ref.^31^	Present Outcomes
						$\tfrac{{\boldsymbol{N}}{{\boldsymbol{u}}}_{{\boldsymbol{x}}}}{\sqrt{{\bf{R}}{{\bf{e}}}_{{\boldsymbol{x}}}}}$	$\tfrac{{\boldsymbol{N}}{{\boldsymbol{u}}}_{{\boldsymbol{x}}}}{\sqrt{{\bf{R}}{{\bf{e}}}_{{\boldsymbol{x}}}}}$
0.1	0.1	0.1	0.1	0.1	0.1	0.8254	0.8254
0.2	0.1	0.1	0.1	0.1	0.1	0.8599	0.8599
0.3	0.1	0.1	0.1	0.1	0.1	0.8948	0.8948
0.1	1.3	0.1	0.1	0.1	0.1	0.8254	0.8254
0.1	1.4	0.1	0.1	0.1	0.1	0.8225	0.8225
0.1	1.5	0.1	0.1	0.1	0.1	0.8200	0.8200
0.1	0.1	0.1	0.1	0.1	0.1	0.8254	0.8254
0.1	0.1	0.2	0.1	0.1	0.1	0.8322	0.8322
0.1	0.1	0.3	0.1	0.1	0.1	0.8387	0.8387
0.1	0.1	0.1	0.6	0.1	0.1	0.7542	0.7542
0.1	0.1	0.1	0.7	0.1	0.1	0.8254	0.8254
0.1	0.1	0.1	0.8	0.1	0.1	0.8930	0.8930
0.1	0.1	0.1	0.1	0.1	0.1	0.8254	0.8254
0.1	0.1	0.1	0.1	0.2	0.1	0.7958	0.7958
0.1	0.1	0.1	0.1	0.3	0.1	0.7662	0.7662
0.1	0.1	0.1	0.1	0.1	0.1	0.8254	0.8254
0.1	0.1	0.1	0.1	0.1	0.2	0.7789	0.7789
0.1	0.1	0.1	0.1	0.1	0.3	0.7275	0.7275

**Table 3:** LSFC comparison with ref.^43^.

*K*	*β*	*λ*_*m*_	Pr	*Sc*	*Q* ^+^	*δ*1	*δ*2	ref.^43^		Present Outcomes	
								*F″*(0)	0.5*C*_*F*_$\sqrt{{\bf{R}}{{\bf{e}}}_{{\boldsymbol{x}}}}$	*F″*(0)	0.5*C*_*F*_$\sqrt{{\bf{R}}{{\bf{e}}}_{{\boldsymbol{x}}}}$
0.2	0.1	0.1	0.1	0.1	0.1	0.1	0.1	−1.0510	−2.1020	−1.0510	−2.1020
0.4	0.1	0.1	0.1	0.1	0.1	0.1	0.1	−1.5047	−3.0094	−1.5047	−3.0094
0.6	0.1	0.1	0.1	0.1	0.1	0.1	0.1	−2.0257	−4.0514	−2.0257	−4.0514
0.1	1.1	0.1	0.1	0.1	0.1	0.1	0.1	−0.8795	−1.7590	−0.8795	−1.7590
0.1	1.2	0.1	0.1	0.1	0.1	0.1	0.1	−0.8920	−1.7840	−0.8920	−1.7840
0.1	1.3	0.1	0.1	0.1	0.1	0.1	0.1	−0.9033	−1.8066	−0.9033	−1.8066
0.1	0.1	0.2	0.1	0.1	0.1	0.1	0.1	−0.8305	−1.6610	−0.8305	−1.6610
0.1	0.1	0.4	0.1	0.1	0.1	0.1	0.1	−0.7616	−1.5232	−0.7616	−1.5232
0.1	0.1	0.6	0.1	0.1	0.1	0.1	0.1	−0.6940	−1.3880	−0.6940	−1.3880
0.1	0.1	0.1	0.3	0.1	0.1	0.1	0.1	−0.8667	−1.7334	−0.8667	−1.7334
0.1	0.1	0.1	0.4	0.1	0.1	0.1	0.1	−0.8690	−1.7380	−0.8690	−1.7380
0.1	0.1	0.1	0.5	0.1	0.1	0.1	0.1	−0.8711	−1.7422	−0.8711	−1.7422
0.1	0.1	0.1	0.1	0.2	0.1	0.1	0.1	−0.8656	−1.7312	−0.8656	−1.7312
0.1	0.1	0.1	0.1	0.4	0.1	0.1	0.1	−0.8658	−1.7316	−0.8658	−1.7316
0.1	0.1	0.1	0.1	0.6	0.1	0.1	0.1	−0.8661	−1.7322	−0.8661	−1.7322
0.1	0.1	0.1	0.1	0.1	0.2	0.1	0.1	−0.8652	−1.7304	−0.8652	−1.7304
0.1	0.1	0.1	0.1	0.1	0.4	0.1	0.1	−0.8650	−1.7300	−0.8650	−1.7300
0.1	0.1	0.1	0.1	0.1	0.6	0.1	0.1	−0.8646	−1.7292	−0.8646	−1.7292
0.1	0.1	0.1	0.1	0.1	0.1	0.2	0.1	−0.8644	−1.7288	−0.8644	−1.7288
0.1	0.1	0.1	0.1	0.1	0.1	0.4	0.1	−0.8728	−1.7456	−0.8728	−1.7456
0.1	0.1	0.1	0.1	0.1	0.1	0.6	0.1	−0.8813	−1.7626	−0.8813	−1.7626
0.1	0.1	0.1	0.1	0.1	0.1	0.1	0.2	−0.8666	−1.7332	−0.8666	−1.7332
0.1	0.1	0.1	0.1	0.1	0.1	0.1	0.4	−0.8614	−1.7228	−0.8614	−1.7228
0.1	0.1	0.1	0.1	0.1	0.1	0.1	0.6	−0.8601	−1.7202	−0.8601	−1.7202

**Table 4:** LNN comparison with ref.^43^.

*K*	*β*	*λ*_*m*_	Pr	*δ*_1_	*Q* ^+^	ref.^43^	Present Outcomes
						$\tfrac{{\boldsymbol{N}}{{\boldsymbol{u}}}_{{\boldsymbol{x}}}}{\sqrt{{\bf{R}}{{\bf{e}}}_{{\boldsymbol{x}}}}}$	$\tfrac{{\boldsymbol{N}}{{\boldsymbol{u}}}_{{\boldsymbol{x}}}}{\sqrt{{\bf{R}}{{\bf{e}}}_{{\boldsymbol{x}}}}}$
0.2	0.1	0.1	0.1	0.1	0.1	0.4260	0.4260
0.4	0.1	0.1	0.1	0.1	0.1	0.5331	0.5331
0.6	0.1	0.1	0.1	0.1	0.1	0.6326	0.6326
0.1	1.1	0.1	0.1	0.1	0.1	0.3667	0.3667
0.1	1.2	0.1	0.1	0.1	0.1	0.3663	0.3663
0.1	1.3	0.1	0.1	0.1	0.1	0.3659	0.3659
0.1	0.1	0.2	0.1	0.1	0.1	0.3684	0.3684
0.1	0.1	0.4	0.1	0.1	0.1	0.3709	0.3709
0.1	0.1	0.6	0.1	0.1	0.1	0.3733	0.3733
0.1	0.1	0.1	0.3	0.1	0.1	0.4223	0.4223
0.1	0.1	0.1	0.4	0.1	0.1	0.4507	0.4507
0.1	0.1	0.1	0.5	0.1	0.1	0.5091	0.5091
0.1	0.1	0.1	0.1	0.2	0.1	0.4059	0.4059
0.1	0.1	0.1	0.1	0.4	0.1	0.4030	0.4030
0.1	0.1	0.1	0.1	0.6	0.1	0.4003	0.4003
0.1	0.1	0.1	0.1	0.1	0.2	0.3947	0.3947
0.1	0.1	0.1	0.1	0.1	0.4	0.3810	0.3810
0.1	0.1	0.1	0.1	0.1	0.6	0.3671	0.3671

**Table 5:** LSN comparison with ref.^43^.

*K*	*Sc*	*δ*_2_	*R*_*c*_	ref.^43^	Present Outcomes
				$\tfrac{{\boldsymbol{S}}{{\boldsymbol{h}}}_{{\boldsymbol{x}}}}{\sqrt{{\bf{R}}{{\bf{e}}}_{{\boldsymbol{x}}}}}$	$\tfrac{{\boldsymbol{S}}{{\boldsymbol{h}}}_{{\boldsymbol{x}}}}{\sqrt{{\bf{R}}{{\bf{e}}}_{{\boldsymbol{x}}}}}$
0.2	0.1	0.1	0.1	0.4702	0.4702
0.3	0.1	0.1	0.1	0.5107	0.5107
0.4	0.1	0.1	0.1	0.5725	0.5725
0.1	0.2	0.1	0.1	0.4860	0.4860
0.1	0.3	0.1	0.1	0.5544	0.5544
0.1	0.4	0.1	0.1	0.6202	0.6202
0.1	0.1	0.2	0.1	0.2434	0.2434
0.1	0.1	0.3	0.1	0.3324	0.3324
0.1	0.1	0.4	0.1	0.4215	0.4215
0.1	0.1	0.1	0.2	0.4213	0.4213
0.1	0.1	0.1	0.3	0.4343	0.4343
0.1	0.1	0.1	0.4	0.4470	0.4470

**Table 6:** LSFC comparison with ref.^45^.

*K*	*β*	*λ*_*m*_	Pr	*F*″(0)	ref.^45^	Present Outcomes
					0.5*C*_*f*_$\sqrt{{\bf{R}}{{\bf{e}}}_{{\boldsymbol{x}}}}$	0.5*C*_*f*_$\sqrt{{\bf{R}}{{\bf{e}}}_{{\boldsymbol{x}}}}$
0.4	0.1	0.1	0.1	−0.7863	−2.3589	−2.3589
0.5	0.1	0.1	0.1	−0.9204	−2.7612	−2.7612
0.6	0.1	0.1	0.1	−1.0539	−3.1617	−3.1617
0.1	1.1	0.1	0.1	−0.7969	−2.3907	−2.3907
0.1	1.2	0.1	0.1	−0.8110	−2.4330	−2.4330
0.1	1.3	0.1	0.1	−0.8236	−2.4708	−2.4708
0.1	0.1	0.2	0.1	−0.4064	−1.2192	−1.2192
0.1	0.1	0.4	0.1	−0.4008	−1.2024	−1.2024
0.1	0.1	0.6	0.1	−0.3953	−1.1859	−1.1859
0.1	0.1	0.1	0.8	−0.4092	−1.2276	−1.2276
0.1	0.1	0.1	1.0	−0.4092	−1.2276	−1.2276
0.1	0.1	0.1	1.2	−0.4092	−1.2276	−1.2276

**Table 7:** LNN comparison with ref. ^45^.

*K*	Pr	*δ*_1_	ref.^45^	ref.^45^	Present Outcomes
			−*T*′(0)	$\tfrac{{\boldsymbol{N}}{{\boldsymbol{u}}}_{{\boldsymbol{x}}}}{\sqrt{{\bf{R}}{{\bf{e}}}_{{\boldsymbol{x}}}}}$	$\tfrac{{\boldsymbol{N}}{{\boldsymbol{u}}}_{{\boldsymbol{x}}}}{\sqrt{{\bf{R}}{{\bf{e}}}_{{\boldsymbol{x}}}}}$
0.3	0.1	0.1	0.7817	1.09438	1.09438
0.5	0.1	0.1	1.1299	1.58186	1.58186
0.7	0.1	0.1	1.4716	2.06024	2.06024
0.1	1.5	0.1	1.4024	1.96336	1.96336
0.1	1.7	0.1	1.4712	2.05968	2.05968
0.1	1.9	0.1	1.5332	2.14648	2.14648
0.1	0.1	0.2	0.2585	0.36190	0.36190
0.1	0.1	0.4	0.1939	0.27146	0.27146
0.1	0.1	0.6	0.1293	0.18102	0.18102

**Table 8:** LSN comparison with ref.^45^.

*K*	*δ*_2_	*Le*	Pr	ref.^45^	Present Outcomes
				$\tfrac{{\boldsymbol{S}}{{\boldsymbol{h}}}_{{\boldsymbol{x}}}}{\sqrt{{\bf{R}}{{\bf{e}}}_{{\boldsymbol{x}}}}}$	$\tfrac{{\boldsymbol{S}}{{\boldsymbol{h}}}_{{\boldsymbol{x}}}}{\sqrt{{\bf{R}}{{\bf{e}}}_{{\boldsymbol{x}}}}}$
0.2	0.1	0.1	0.1	1.4913	1.4913
0.3	0.1	0.1	0.1	1.8466	1.8466
0.4	0.1	0.1	0.1	2.1845	2.1845
0.1	0.1	0.1	0.1	1.1297	1.1297
0.1	0.2	0.1	0.1	1.0858	1.0858
0.1	0.3	0.1	0.1	1.0420	1.0420
0.1	0.1	0.4	0.1	1.1546	1.1546
0.1	0.1	0.5	0.1	1.1627	1.1627
0.1	0.1	0.6	0.1	1.1706	1.1706
0.1	0.1	0.1	1.3	1.4752	1.4752
0.1	0.1	0.1	1.5	1.5020	1.5020
0.1	0.1	0.1	1.7	1.5271	1.5271

### Discussion

The generalized flow problem for the Casson fluid flow around a cylindrical geometry is controlled by mathematical formulation given in Eqs (2–6) in terms of partial differential equations. For solution purpose an acceptable set of transformation given in Eq. (7) is used to reduce partial differential equations into set of coupled ordinary differential equations. Since the reduced problem is boundary value problem therefore to meet the criteria of computational algorithm a dummy substitution is introduced and initial value problem is established. A shooting method along with Runge-Kutta scheme is implemented and the obtained observations are offered in terms of both tabular and graphical trends. One can easily check that our results are in excellent match with existing literature subject to local skin friction coefficient, local Nusselt and Sherwood numbers, see all of the tables in this regard. The stream lines topologies for the Casson liquid towards various involved physical parameters namely, magnetic field parameter (*γ*), curvature parameter (*K*), velocity ration parameter (*A*) and Casson fluid parameter (*β*) are presented. In detail, Figs 3 and 4 reports the stream lines patterns of Casson fluid in a magnetized (*γ* ≠ 0) doubly stratified (*δ*_1_ ≠ 0, *δ*_2_ ≠ 0) medium for both cylindrical (*K* ≠ 0) and flat (*K* = 0) surfaces. Particularly, Fig. 3 shows the stream lines pattern for cylindrical surface when *K* = 0.7 and Fig. 4 is examine for flat surface that is *K* = 0.0. Both patterns depicts magnetized stratified medium for Casson fluid and one can observed that the streamlines for *γ* = 0.5, *K* = 0.7 are significantly close as compared to *γ* = 0.5, *K* = 0.0. This fact is due to cylindrical surface and hence Casson fluid velocity enlarged for the case of cylindrical geometry with respect to flat surface even the fluid flow regime is magnetized. Figures 5 and 6 provided stream lines topologies when fluid flow regime is independent of applied magnetic field. It is observed that in non-magnetized stratified medium the stream lines are enough closer for cylindrical surface as compared to flat plate. Moreover, one can validated from Figs 3 and 4 being plotted in magnetized stratified medium (*γ* ≠ 0) in contrast to non-magnetized stratified medium (*γ* = 0) In this direction it is observed that the existence of magnetic field is the cause of wider distance between stream lines (Figs 3 and 4) with that of stream lines patterns (Figs 5 and 6) for without the influence of magnetic field. Therefore, a turbulence character can be avoid by considering magnetized flow field. In general, the presence of magnetic field parameter is the cause of increment in Lorentz force. It is resistive force and may cause resistance to fluid flow as a result the average velocity diminishing. The variations in Casson fluid velocity under the region of stagnation point for both magnetized and non-magnetized stratified medium is tested for (*A* < 1, *A* = 1, *A* > 1). The Figs 7–12 are provided in this direction. Figures 7 and 8 are plotted for *A* < 1, when the free stream velocity is lower in strength as compared to stretching rate in both magnetized and non-magnetized stratified medium. One can observed that the for magnetized case *γ* = 0.5 the stream lines patterns are wider than that of non-magnetized case *γ* = 0.0 as expected. The case *A* = 1, when both free stream and stretching velocities are equivalent is examined and observation in this regard is provided by means of Figs 9 and 10 for both magnetized and non-magnetized frame. In magnetized frame the velocity seems suppressed than that of non-magnetized. This is due to opposing force termed as Lorentz force. When free stream velocity exceeded the strength with that of stretching one we interpret this with *A* > 1. Figures 11 and 12 are given with respect to *A* > 1 in both magnetized and non-magnetized stratified medium. It can be seen that for *A* > 1 the impact of magnetic field parameter still remains significant that is the free stream lines patterns depicts larger distance between them as compared to non-magnetized frame which implies the suppress nature of velocity against magnetic flow field. Figures 13 and 14 are plotted to report flow pattern of Casson liquid (*β* = 1.3) under the region of stagnation point (*A* ≠ 0) flow in both magnetized (*γ* = 0.5) and non-magnetized (*γ* = 0) stratified medium. It is seen that the Casson fluid particles momentum is significant in non-magnetized (*γ* = 0) stratified medium than that of magnetized (*γ* = 0.5). The application of magnetic field brings the resistive role of Lorentz force as results the average velocity of Casson liquid suffers. It can be observed from Eq. (9), when Casson fluid parameters approaches the infinite value (*β* → ∞) we have viscous fluid via doubly stratified medium having suspended nanoparticles in flow regime. Therefore, Figs 15 and 16 reports the flow pattern of viscous fluid in both in both magnetized (*γ* = 0.5) and non-magnetized (*γ* = 0) stratified medium. Similar trends are observed for the existing of magnetized stratified medium with that of non-magnetized frame. The viscous fluid momentum is decreasing function of magnetic field parameter. On another hand when the Casson fluid parameter increases the fluid becomes more dense as a result the velocity increases. This fact is validated through stream lines patterns that is for *β* → 1.3 the distance between stream lines are less as compared to stream lines pattern given in Fig. 16. The extreme limit of Casson fluid parameter (*β* → ∞) yields Newtonian fluid model as an especial case.

## Graphical outcomes

Additionally, Figs 17–20 are plotted to provide the impact of flow controlling parameters namely, mixed convection parameter (*λ*_*m*_), thermal radiation parameter (*R*_*T*_), temperature stratification parameter (*δ*_1_) and chemical reaction parameter (*R*_*c*_). To be more specific, the impact of mixed convection parameter on fluid velocity is examined and provided by way of Fig. 17. It is noticed that when we iterate *λ*_*m*_ = 0.1, 0.3, 0.4 the fluid velocity enhances. Figure 18 is plotted to offer the effect of thermal radiation parameter on fluid temperature. It can be seen that when we iterate *R*_*T*_ = 0.1, 0.2, 0.4 the fluid temperature shows an inciting values. Further, it is noticed that the fluid temperature is decreasing function of temperature stratification parameter. Figure 19 is evident in this direction that is for positive values of *δ*_1_ = 0.1, 0.2, 0.3 the fluid temperature reflects decline nature. The effect of chemical reaction parameter on nano-concentration profile is examined and offered by means of Fig. 20. It is observed that the nano-concentration profile is decreasing function of *R*_*c*_ = 0.1, 0.3, 0.4.

## Sub attempts

Since an article contains proposed generalized mathematical modelling for the stratified medium towards Casson fluid flow around a cylindrical geometry. The existing literature subject to the Casson flow field via stratified medium becomes over sub-study. The detail in this direction structured through section wise.

### Case-1

In the absence of concentration equation and ignoring the stagnation point, thermal radiations, Joule heating and suspended nanoparticles assumptions, the generalized problem reduced to Problem reported in ref.^31^. In this problem a magnetohydrodynamic Casson fluid flow towards both flat and cylindrical surfaces via thermally stratified medium. It was concluded that the obtained variations were remarkably enormous for cylindrical geometry as compared to plane surface. The local skin friction coefficient was found as an increasing function of curvature parameter, Casson fluid parameter, Prandtl number and thermal stratification parameter while opposite trends were noticed towards mixed convection parameter and heat generation parameter. The local Nusselt number was an increasing function for greater values of curvature parameter, mixed convection parameter and Prandtl number while inverse values are noticed for Casson fluid parameter, thermal stratification parameter and heat generation parameter. An excellent comparison is noticed with ref.^31^ for both local skin friction coefficient and local Nusselt number. Tables 1 and 2 are constructed in this regard. The flow narrating formulation in this regard is given as:

18 $$\begin{array}{c}(1+2K\eta )\frac{{d}^{3}F(\eta )}{d{\eta }^{3}}+2K\frac{{d}^{2}F(\eta )}{d{\eta }^{2}}+\frac{1}{\beta }((1+2K\eta )\frac{{d}^{3}F(\eta )}{d{\eta }^{3}}+2K\frac{{d}^{2}F(\eta )}{d{\eta }^{2}})\\ \,\,\,+F(\eta )\frac{{d}^{2}F(\eta )}{d{\eta }^{2}}-{(\frac{dF(\eta )}{d\eta })}^{2}-{\gamma }^{2}(\frac{dF(\eta )}{d\eta })+{\lambda }_{m}T(\eta )=0,\end{array}$$

19 $$(1+2K\eta )\frac{{d}^{2}T(\eta )}{d{\eta }^{2}}+2K\frac{dT(\eta )}{d\eta }+{\rm{\Pr }}(F(\eta )\frac{dT(\eta )}{d\eta }-\frac{dF(\eta )}{d\eta }{\delta }_{1}+QT(\eta )-\frac{dF(\eta )}{d\eta }T(\eta ))=0,$$

20 $$\begin{array}{c}\frac{dF(\eta )}{d\eta }=1,\,\,F(\eta )=0,\,\,T(\eta )=1-{\delta }_{1},\,\,{\rm{at}}\,\,\eta =0,\\ \frac{dF(\eta )}{d\eta }\to 0,\,\,T(\eta )\to 0,\,\,{\rm{when}}\,\eta \to \infty .\end{array}$$

### Case-2

In absence of thermal radiations, Joule heating, suspended nanoparticles and stagnation point assumptions our generalized mathematical formulation meet with formulation reported in ref.^43^. In this problem doubly stratified mixed convection flow of Casson fluid due to an inclined cylindrical surface was examined. It was reported that in absolute sense the local skin friction shows an inciting nature towards curvature parameter, Casson fluid parameter, Prandtl number, Schmidt number, thermal stratification parameter while opposite attitude wasobserved for the positive values of mixed convection parameter, solutal stratification parameter and heat generation parameter. It was seen that Nusselt number was decreasing function of Casson fluid parameter, thermal stratification parameter and heat generation parameter but an inverse trends were found for the higher values of mixed convection parameter, curvature parameter and Prandtl number. Additionally, it was observed that Sherwood number reflected an inciting attitude for higher values of curvature parameter, Schmidt number, solutal stratification parameter and chemical reaction parameter. Tables 3, 4 and 5 are constructed in this direction and we have an excellent match with observation recorded in ref. ^43^. The corresponding mathematical formulation are prearranged as follows:

21 $$\begin{array}{c}(1+2K\eta )\frac{{d}^{3}F(\eta )}{d{\eta }^{3}}+2K\frac{{d}^{2}F(\eta )}{d{\eta }^{2}}+\frac{1}{\beta }((1+2K\eta )\frac{{d}^{3}F(\eta )}{d{\eta }^{3}}+2K\frac{{d}^{2}F(\eta )}{d{\eta }^{2}})\\ \,\,\,+\,F(\eta )\frac{{d}^{2}F(\eta )}{d{\eta }^{2}}-{(\frac{dF(\eta )}{d\eta })}^{2}-{\gamma }^{2}(\frac{dF(\eta )}{d\eta })+{\lambda }_{m}(T(\eta )+NC(\eta ))\cos \,\alpha =0,\end{array}$$

22 $$(1+2K\eta )\frac{{d}^{2}T(\eta )}{d{\eta }^{2}}+2K\frac{dT(\eta )}{d\eta }+{\rm{\Pr }}(F(\eta )\frac{dT(\eta )}{d\eta }-\frac{dF(\eta )}{d\eta }{\delta }_{1}+QT(\eta )-\frac{dF(\eta )}{d\eta }T(\eta ))=0,$$

23 $$(1+2K\eta )\frac{{d}^{2}C(\eta )}{d{\eta }^{2}}+Sc(F(\eta )\frac{dC(\eta )}{d\eta }-\frac{dF(\eta )}{d\eta }C(\eta )-\frac{dF(\eta )}{d\eta }{\delta }_{2}-{R}_{c}C(\eta ))=0,$$

with transformed conditions:

24 $$\begin{array}{c}\frac{dF(\eta )}{d\eta }=1,\,\,F(\eta )=0,\,\,T(\eta )=1-{\delta }_{1},\,\,C(\eta )=1-{\delta }_{2},\,{\rm{at}}\,\eta =0,\\ \frac{dF(\eta )}{d\eta }\to 0,\,\,T(\eta )\to 0,\,\,C(\eta )\to 0,\,{\rm{when}}\,\eta \to \infty .\end{array}$$

### Case-3

One can confirm from mathematical formulation given by Eqs (9–12), that the generalized problem meets with ref.^44^ when we ignore stagnation point, magnetic field, suspended nanoparticles, thermal radiations, heat generation and Joule heating effects along additional condition that is *β* → ∞. In this problem mixed convection effects towards thermally stratified media were presented. The corresponding mathematical formulation in this problem is as follows:

25 $$(1+2K\eta )\frac{{d}^{3}F(\eta )}{d{\eta }^{3}}+2K\frac{{d}^{2}F(\eta )}{d{\eta }^{2}}+F(\eta )\frac{{d}^{2}F(\eta )}{d{\eta }^{2}}-{(\frac{dF(\eta )}{d\eta })}^{2}+{\lambda }_{m}(T(\eta ))=0,$$

26 $$(1+2K\eta )\frac{{d}^{2}T(\eta )}{d{\eta }^{2}}+2K\frac{dT(\eta )}{d\eta }+{\rm{\Pr }}(F(\eta )\frac{dT(\eta )}{d\eta }-\frac{dF(\eta )}{d\eta }{\delta }_{1}-\frac{dF(\eta )}{d\eta }T(\eta ))=0,$$

with transformed conditions:

27 $$\begin{array}{c}\frac{dF(\eta )}{d\eta }=1,\,\,F(\eta )=0,\,\,T(\eta )=1-{\delta }_{1},\,\,{\rm{at}}\,\eta =0,\\ \frac{dF(\eta )}{d\eta }\to 0,\,\,T(\eta )\to 0,\,\,{\rm{when}}\,\eta \to \infty .\end{array}$$

### Case-4

The effectiveness subject to velocity gradients due to viscous stresses was consider very small therefore in this attempt^45^ the impact of Joule heating was ignored. Since our flow problem contains the influence of Joule heating so by ignoring this fact our findings exactly matched with results reported in ref.^45^. For comparison purpose we have constructed Tables 6, 7 and 8. To be more specific, Table 6 is constructed for comparative values of local skin friction coefficient. It was found In absolute sense, it was noticed that the skin friction coefficient reflected increasing values via both *K*, and *β*. On the other hand it shows an opposite trends for the increasing values of *λ*_*m*_. Moreover, the local skin friction coefficient in independent of Prandtl number. The negative sign of local skin friction implies the drag forced exerted by cylindrical surface towards Casson fluid particles. It has been observed through the local Nusselt number shows an increasing values for the greater values of *K* and Pr, however opposite variations are observed for the increasing values of the parameter *δ*_1_. The negative sign in local Nusselt number relates the transfer of heat normal to the cylindrical surface. The local Sherwood number depicts an increasing attitude for the increasing values of the parameters *K*, and *Le*. On the other hand, it shows a decreasing nature towards *δ*_2_. Further, the recent developments on the surface quantities can be assessed in refs^46,47^.

## Summary

The realistic Casson fluid model via both magnetized (*γ* ≠ 0) and non-magnetized (*γ* = 0) stratified (*δ*_1_ ≠ 0, *δ*_2_ ≠ 0) media is investigated. The stream lines topologies are limited to magnetic field parameter (*γ*), curvature parameter (*K*), velocity ratio parameter (*A*) and Casson fluid parameter (*β*). The key outcomes are itemized as follows:

1. A generalized mathematical formulation is proposed for the non-Newtonian fluid model.
2. The Casson liquid around cylindrical surface (*K* = 0.7) claims larger momentum values with respect to Casson liquid via flat surface (*K* = 0.0). This variation is invariant subject to both magnetized and non-magnetized stratified medium.
3. The flow momentum of Casson liquid found suppress for magnetized stratified medium as compared to non-magnetized medium.
4. Both Newtonian (*β* → ∞) and non-Newtonian (*β* = 1.3) fluids flow reflects decline nature in magnetized (*γ* ≠ 0) stratified (*δ*_1_ ≠ 0, *δ*_2_ ≠ 0) medium than that of non-magnetized (*γ* = 0) one.
5. The momentum of viscous fluid flow in stratified medium is significantly lower as compared to momentum of Casson fluid flow. Such variation holds in both magnetized and non-magnetized stratified medium.
6. The Casson fluid temperature is increasing function of thermal radiation parameter.
7. The nano-concentration profile is decreasing function of chemical reaction parameter.
8. The LSFC, LNN and LSN are offered in a systematic design via comparison with some interesting existing results.
